# Network Perspectives on Epilepsy Using EEG/MEG Source Connectivity

**DOI:** 10.3389/fneur.2019.00721

**Published:** 2019-07-17

**Authors:** Pieter van Mierlo, Yvonne Höller, Niels K. Focke, Serge Vulliemoz

**Affiliations:** ^1^Medical Image and Signal Processing Group, Ghent University, Ghent, Belgium; ^2^Faculty of Psychology, University of Akureyri, Akureyri, Iceland; ^3^Clinical Neurophysiology, University Medicine Göttingen, Göttingen, Germany; ^4^EEG and Epilepsy Unit, University Hospital of Geneva, Geneva, Switzerland

**Keywords:** EEG/MEG source connectivity, epilepsy, interictal epileptiform discharges, seizures, resting state

## Abstract

The evolution of EEG/MEG source connectivity is both, a promising, and controversial advance in the characterization of epileptic brain activity. In this narrative review we elucidate the potential of this technology to provide an intuitive view of the epileptic network at its origin, the different brain regions involved in the epilepsy, without the limitation of electrodes at the scalp level. Several studies have confirmed the added value of using source connectivity to localize the seizure onset zone and irritative zone or to quantify the propagation of epileptic activity over time. It has been shown in pilot studies that source connectivity has the potential to obtain prognostic correlates, to assist in the diagnosis of the epilepsy type even in the absence of visually noticeable epileptic activity in the EEG/MEG, and to predict treatment outcome. Nevertheless, prospective validation studies in large and heterogeneous patient cohorts are still lacking and are needed to bring these techniques into clinical use. Moreover, the methodological approach is challenging, with several poorly examined parameters that most likely impact the resulting network patterns. These fundamental challenges affect all potential applications of EEG/MEG source connectivity analysis, be it in a resting, spiking, or ictal state, and also its application to cognitive activation of the eloquent area in presurgical evaluation. However, such method can allow unique insights into physiological and pathological brain functions and have great potential in (clinical) neuroscience.

## Introduction

Epilepsy is commonly considered an archetypical network disease (1), with seizures and interictal activity generated and spreading in networks involving one or both hemispheres. There is a growing body of imaging evidence suggesting that epilepsy affects both structural (2, 3) and functional brain network properties (4–7). Interestingly, even in idiopathic/genetic generalized epilepsy, there is a certain level of focality both in resting-state imaging (7) as well as for generators of epileptiform activity (8) and seizures (9, 10). These structural and functional network properties are investigated using brain connectivity analysis.

Brain connectivity can be categorized into structural, functional and effective connectivity (11). Structural connectivity refers to the white matter connections in the brain and can be examined *in vivo* with MRI measuring the motion of water along the axons. Functional and effective connectivity entangle the neuronal communication between brain regions. These types of connectivity can be calculated when signals are sampled over multiple time points, such as brain activity recorded via EEG, MEG, but also fMRI, or PET. According to Friston (12), “functional connectivity is defined as statistical dependencies among remote neurophysiological events, while effective connectivity refers explicitly to the influence that one neural system exerts over another, either at a synaptic or population level.” Functional or effective connectivity is measured in terms of similarities between signals and shows complementary information with regard to structural connectivity (13). With the growing enthusiasm for connectivity it is often overlooked that in reality, all we have are statistical interdependencies of signals, which should be interpreted cautiously.

The main difference between functional and effective connectivity is that functional connectivity characterizes whether the activity of two units are linked, while effective connectivity also examines the direction of communication, i.e., which is the driver or receiver of information. This does not tell us whether there exists a physical/structural connection (14), but the size of the predictability lets us estimate how likely it is that one unit influences the other. Therefore, the common understanding is that effective connectivity entails directed information flow from one system/region to another while functional connectivity assesses undirected information flow. Functional or effective connectivity can be time-resolved or averaged over a certain period and optionally within a specific frequency band.

The most classical measures for functional connectivity are correlation and coherence, which reflects the similarity between signals in the time and frequency domain, respectively. Intuitively speaking, coherence is a correlation of two signals in the frequency domain (15). Other connectivity measures consider the phase of the oscillations in the electrophysiological signals, the so-called measures of synchronization. The phase indicates whether the oscillation is at a specific time point *t* at a peak, trough, or transitions between these two states (such as for instance, zero crossings). If two signals exhibit the same phases at the same point in time, they are said to oscillate synchronously. Determining the phase of two signals allows calculating the difference in phase, the phase lag, which in turn may inform us about propagation effects, if the one signal exhibits a later phase than the other signal. The phase lag is suggested to reflect signal propagation and can be studied to examine effective connectivity. In addition to bivariate measures that consider pairs of signals, multivariate measures are designed in order to remove shared properties between multiple signals, such as, for example, partial coherence (16). Most measures of effective connectivity are described under the umbrella term Granger causality (17). This concept considers two signals *X* and *Y* and examines whether the activity at time point *t* of signal *X* can be predicted (statistically) by the activity at the earlier time points *t-k* of signal *Y*. Among them, partial directed coherence (18) and directed transfer function (19) are commonly used to study epilepsy. Next to these data driven analysis approaches, effective connectivity can also be estimated based on underlying biophysical models with a priori assumptions about the organization of the network as in Dynamic Causal Modeling (DCM) and other neural mass models. DCM in EEG or MEG takes biologically plausibility of causal models into account, and thus yields an informed estimate of connectivity (20).

The connectome estimated using functional or effective connectivity algorithms contains a large amount of data, which can complicate the biophysical interpretation. The connectome is composed of values indicating the relatedness of each region-by-region combination. In addition, each of these values can be estimated optionally for defined time-windows and/or frequency ranges. That is, the final result may characterize the data in up to four dimensions: region × region × time × frequency. In order to reduce the dimensionality of these complex data and to extract meaningful patterns, topological graph analysis can be performed. The importance of specific nodes and the general architecture can be characterized by local and global network characteristics (21). Local indices identify important information hubs, which distribute or merge information to a large number of other nodes, or select so-called rich-clubs of highly interconnected nodes. Global indices measure the organization of the network into small worlds where only neighboring regions exchange information, or whether the network is organized in a centralized way. Regions with high outflow are considered drivers of information transfer in the network. Efficient information transfer across the network is measured by efficiency or path length. Segregation characterized by groups of highly connected regions for specialization can be measured by the clustering coefficient (22). The clear advantage of deriving network characteristics is that it reduces the statistical challenge posed by the multidimensional problem in terms of multiple comparisons. High dimensional data can lead to false discoveries when the statistical approach does not deal adequately with the high dimensionality. However, a large degree of integration can obscure localized phenomena. Therefore, the degree of integration needs to be chosen carefully in line with the current clinical problem or research question.

Functional and effective connectivity are commonly used to gain insight into the network nature of epilepsy (23). On the one hand, connectivity analysis is used to identify how epilepsy and years of seizures and/or interictal activity affect the brain network (24). Furthermore, cognitive improvement or decline can be linked to changes in specific brain networks in epilepsy patients (25). On the other hand, because seizures and spikes spread rapidly in the brain, connectivity analysis is used as a tool to localize the seizure onset zone (SOZ) and the irritative zone (IZ) (26). Here, a big advantage is that non-invasive connectivity analysis can be validated based on resections that rendered the patients seizure free or intracranial EEG recordings that are often available in these patients.

## MEG/EEG Source Connectivity

The electrical activity of active neurons can be recorded at the scalp surface as voltage differences across EEG electrodes. In addition, the neuronal currents in the brain generate magnetic fields that can be measured outside the scalp surface by the MEG sensors. Compared to other neuroimaging techniques such as PET and fMRI, EEG, and MEG have a superior temporal resolution but an inferior spatial resolution. Despite this inferior spatial resolution, the temporal resolution and the fact that EEG and MEG directly measures neuronal activity makes them highly valuable techniques to study functional and especially effective connectivity. Combining neuroimaging techniques with high spatial resolution with a technique with high temporal resolution, such as EEG-fMRI, is a valid approach to examine slow changes in blood supply based on spiking activity (27–29) or at rest (30) and, therefore, provides an excellent validation for localization accuracy of source connectivity (31). Nevertheless, because MRI induces artifacts such as ballistocardiographic artifact, the EEG signals recorded within the MRI scanner are more noisy and therefore less suitable to be used for connectivity analysis compared to EEG measured outside the MRI scanner.

There are a number of studies applying connectivity algorithms to the electrophysiological signals recorded from the MEG/EEG sensors, so called functional and effective connectivity in sensor space. While this is the most straightforward way to estimate connectivity, this approach suffers from important methodological limitations and these studies should be interpreted with caution. First, due to the volume conduction effect, every source in the brain, i.e., activity in a brain region large enough to generate measurable EEG/MEG signals, is picked up by all recording EEG/MEG sensors simultaneously (32). The main orientation of the activated neurons, the distance to the sensors and the conductive properties of the tissue define how much each source is picked up by each sensor. Because of this volume conduction effect, connectivity analysis performed in sensor space can potentially lead to false connections, given the fact that distant electrode can share common information from several sources (33, 34). Second, the choice of the EEG reference can influence the estimated functional integration and segregation (35). Therefore, the connectivity pattern derived in sensor space does not necessarily reflect the connections of underlying cortical regions. One (partial) solution is to apply connectivity measures to the sensor signals that are specifically designed to deal with the volume conduction effect such as the imaginary part of the coherence (36). Another option to circumvent the volume conduction effect is to study the EEG/MEG signals in source space (37). First, EEG source imaging is performed on the signals to project them into brain/source space. It is of utmost importance to choose a proper forward model and inverse technique to estimate the neuronal activity in the volumes of interest. Even in source space it is important to choose the measure for connectivity carefully, otherwise the artifacts of volume conduction may still be present even in reconstructed source time series as zero-lag correlations (33, 37). Nevertheless, it is an advantage that source space connectivity is a more direct representation of the connectivity pattern between brain regions instead of electrodes as this clearly augments the information gain for clinical and research questions concerning epilepsy. This makes the information from source space connectivity analysis much more intuitive to understand.

**Figure 1:**
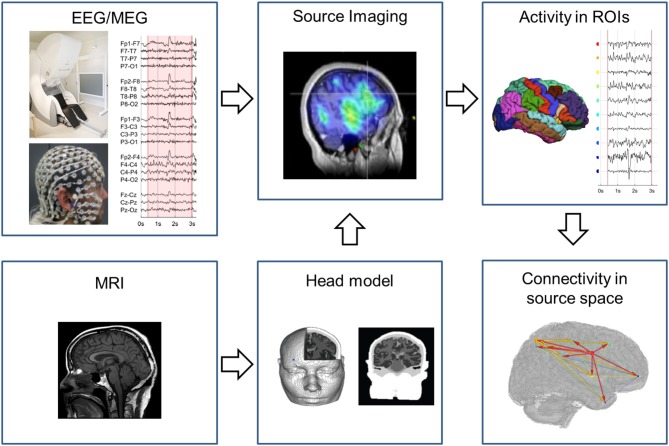
Pipeline to obtain EEG/MEG source connectivity. The EEG/MEG signals in sensor space are source imaged using a head model constructed based on a template or patient specific MRI. In the brain regions of interest the neuronal activity is estimated over time and fed in the connectivity analysis to obtain the connectivity pattern in source space.

In the following, we provide a brief explanation on how EEG/MEG source space connectivity can be calculated from EEG and MEG measurements. In Figure 1 an example pipeline how to extract source space connectivity patterns from EEG/MEG is depicted. In a first step, EEG/MEG sensor signals are recorded. In this step, it is important to assure optimal quality of the measurements. These recordings are then usually preprocessed to remove environmental and physiological noise. For example, this is done by excluding segments of data contaminated by eye or muscle artifacts. Decomposition techniques such as independent component analysis (ICA) are frequently used to remove eye blinks or cardiac artifacts. However, one should be cautious since ICA might introduce spurious connectivity as it removes shared activity from all sensors. In other words, ICA employs a calculation on all channels that might introduce similarity between the signals. The effects of ICA artifact removal on subsequent connectivity analyses is not clearly quantified yet and should be clarified in future studies. Also filtering of the EEG and MEG signals should be done carefully in order to prevent the introduction of phase differences. Therefore, it is recommended to use zero-phase shift filters, for instance, to remove 50/60 Hz power line noise and to refrain from filtering as much as possible (38).

In a second step, the EEG/MEG signals are projected from sensor to source space using EEG/MEG source imaging (ESI/MSI). ESI/MSI is applied to all time points of the chosen pre-processed epoch. For each time point a source image is generated. From the source images, the activity in the ROIs that are defined based on a cortical parcellation (based on an atlas or specifically defined for the study) can be estimated over time. It is also possible to reconstruct EEG/MEG time courses on a voxel- or vertex-level in higher spatial resolution, although this increases the computational resources needed and is limited by the spatial resolution of modalities. M/ESI requires a forward model that characterizes the electrical and magnetic field propagation in the subject's head. From the individual's MRI, this electromagnetic head model is constructed that links brain activity to the recorded scalp potentials. For EEG the use of a complex head model that specifies each tissue class is recommended, while for MEG simpler models usually suffice. This is because the spread of magnetic fields are not affected by electric conductance, in contrary to electric fields; i.e., the complex architecture of the head, including cerebrospinal fluid, dura, skull, fatty tissue, and skin, all of which having different conductance, does not influence magnetic fields (39). This enables the use of much simpler head models in MEG, even the very simple “single shell” model are still frequently used (40, 41). Nevertheless, it has been shown that not including CSF and not distinguishing gray and white matter in the head models can introduce source space connectivity errors both in EEG and MEG (42). The inverse solution techniques depend on the forward model to estimate the neuronal activity from the M/EEG. These head models can be divided into dipole modeling techniques, where the number of estimated sources in the brain is much smaller than the number of sensors, and distributed inverse solution techniques that estimate the activity in many sources in the brain using different methods of regularization (43). Most commonly used inverse techniques for M/EEG source space connectivity are distributed techniques such as weighted minimum norm, beamforming and low resolution brain electromagnetic tomography (LORETA). It has been shown that the optimal choice of the inverse solution depends both on the spatial and synchronization profile of the interacting cortical sources (44). Also, the intrinsic difference between EEG and MEG influences the result. MEG is particularly sensitive to tangential dipoles, whereas it is “blind” to pure radial sources, while the EEG is more sensitive to radial sources (45, 46). Hence, one could expect that some source connections are particularly well detectable with MEG (if they are largely tangential to the skull convexity), whereas others should be better detectable with EEG or a combination of both techniques.

In a last step, connectivity measures, as introduced above, can be applied to the estimated neuronal activity in the ROIs to obtain the connectivity pattern in source space. Graph analysis can be applied on this connectome to extract local and global characteristics of the network.

For source-level analysis of EEG/MEG signals several non-commercial software packages offer tools and functions. For example, EEGLAB (47), CARTOOL (48), Fieldtrip (49), Brainstorm (50), eConnectome (51), and the MNE software (52) offer both, source-transformation and connectivity analysis.

## Source Connectomes in Epilepsy

In this section, we provide an overview how EEG/MEG source space connectivity has been used in epilepsy patients to study ictal, interictal, and resting state activity.

### Ictal

Ding et al. studied EEG source space connectivity in 20 seizures of 5 patients (53). The brain source with highest outgoing information flow was estimated within 15 mm of the EZ that was defined by lesions visible on MRI or hyper perfusion seen in ictal SPECT. Given that the resection location in the patients was not mentioned, it remains unclear if the presumed EZ corresponded with the true EZ. In a follow-up study, Lu et al. (54) showed the value of using more electrodes to localize the SOZ based on EEG source space connectivity by comparing different electrode setups (76, 64, 48, 32, and 21 electrodes). In the 10 investigated patients with ILAE class 1 or 2 post-operative outcome, the SOZ was estimated within 10 mm of the resection in 16/23 seizures and within 20 mm in 22/23 seizures. The gain in sensitivity to localize the SOZ when increasing the number of scalp electrodes has been confirmed by Staljanssens et al. (26). However, the study compared the same data with the full number of sponge-electrodes (265) from a high-impedance amplifier to a reduced subset (reduced sequentially up to 32 channels). The drawback of this approach is that low-density systems typically used in a clinical setting have different amplifiers and electrodes, such that the data quality is not directly comparable between both situation and therefore the result of reducing the number of electrodes should be interpreted with caution.

Currently, the recording length using high density EEG is limited to overnight recordings, at most. The resulting difficulty to record seizures with high density EEG pushed for ictal connectivity analysis based on clinical video-EEG. The largest cohort study so far was performed by Staljanssens et al. (55). One hundred and eleven seizures in 27 patients all with Engel class 1 outcome were localized using EEG source connectivity and ESI power. They showed that source space connectivity, compared to ESI power, significantly increases the performance from 42 to 94% to localize the SOZ within 10 mm from the resection. Despite the fact that several studies (26, 53–57) show the potential of ictal source localization using EEG source connectivity, there is only limited data available about their sensitivity and specificity in extra-temporal lobe epilepsy or in patients that did not become seizure free, which hampers the use of these methods in a clinical setting.

**Figure 2:**
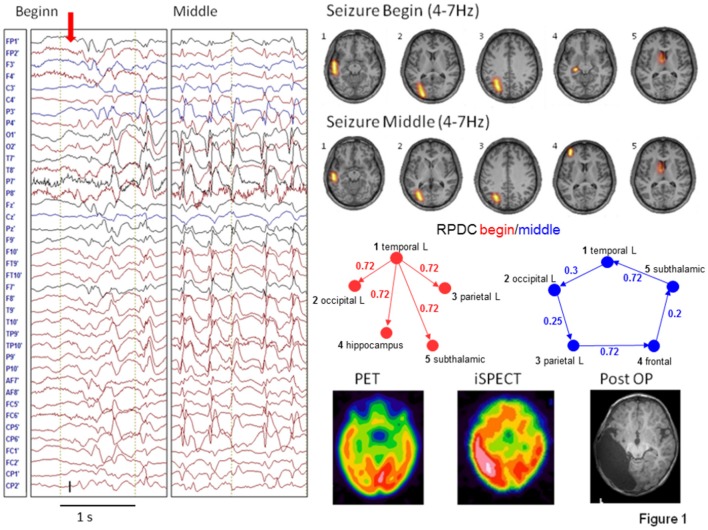
Figure reproduced from Elshoff et al. (58). In the beginning of the seizure a star-shaped network topology with the SOZ as main hub is found, while during the middle of the seizure a circular network was found. Permission granted to reproduce under the terms of the Creative Commons Attribution License.

EEG source connectivity has also been used to investigate the network topology during a seizure as a marker of transient functional reorganization. Elshoff et al. showed that the topology changes from a star-like topography with the SOZ as the main hub in the beginning of the seizure to a circular pattern with no hub in the middle of the seizure (Figure 2). These results suggest an important information transfer from the SOZ at seizure onset, that was reduced during the seizure and resulted in a reduction of the efficiency of information transfer (58). Japaridze et al. used the same approach in 15 children with continuous spike waves during sleep and reported network abnormalities involving notably the thalamus although the possibility of EEG to estimate activity in the thalamus remains very questionable (59). Klamer et al. studied seizures and auras of a patient with musicogenic epilepsy using DCM based on prior selection of regions of interest from fMRI (60). In this application, the technique was used to infer hidden neuronal states from measurements of brain activity, to localize the SOZ from simultaneous high density EEG/MEG. Two sources were apparent from previous functional MRI investigation: one frontal and one mesiotemporal. Using DCM they found that the best model explaining the recording consisted in the mesiotemporal brain region driving the frontal regions. In later invasive EEG recordings the right hippocampus was confirmed as SOZ. It is important to note that the regions of interest were selected based on results of the fMRI analysis and on previous literature; without these priors, the results of the source connectivity analysis might have been considerably different.

### Interictal Epileptic Discharges

EEG and MEG are sensitive to different type of spikes. EEG is more sensitive to spikes arising from cortex in which the pyramidal neurons are aligned perpendicular to the skull, while MEG is more sensitive to tangential sources. One recent study reported that about 8% of spike types (from ~300 patients) were only visible in MEG (61), whereas another study reported an added sensitivity of 18% for MEG vs. EEG in 22 epilepsy patients (62). The problem with all studies is that EEG and MEG channel count and coverage are often not directly comparable. MEG usually has >250 sensors, while EEG is often recorded with ~1/10 of these. A head-to-head comparison of MEG and high density EEG is still lacking.

M/ESI applied to interictal spikes has been increasingly validated by large independent studies showing its accuracy as a surrogate marker of epileptic activity. M/ESI has high sensitivity and specificity to predict epilepsy surgery outcome by localizing the irritative zone that generates the spikes (63–66). The feasibility to use MEG source connectivity to localize spikes has been shown by Dai et al. (67). Dai et al. selected time points of interest visually and, from these selected epochs, regions of interest that—according to visual inspection—exhibited significant activity. We stress that, in order to improve objectivity of source connectivity, it is absolutely necessary to follow standardized rules or statistical approaches to select regions of interest.

Storti et al. (68) performed source analysis and Granger-based connectivity on high density EEG in 12 patients with focal epilepsy. They found that connectivity could distinguish between spike onset and propagation zones. In this study, only half of the patients were operated and post-operatively seizure free so that validation was only partially available.

**Figure 3:**
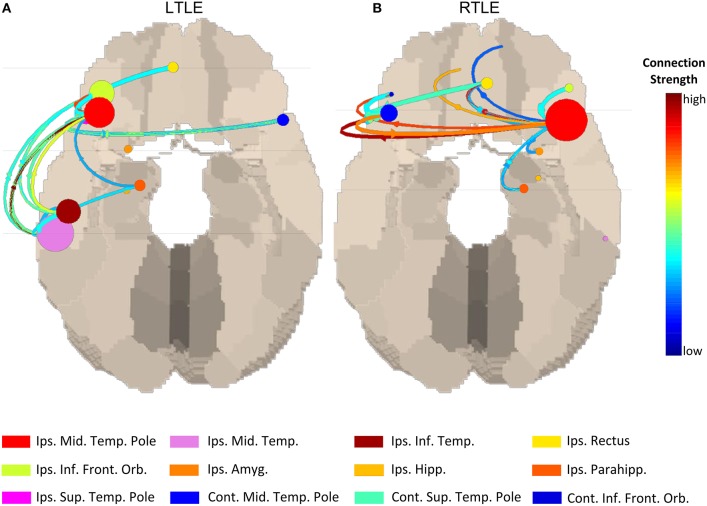
Source connectivity pattern during interictal spikes at group level left temporal lobe epilepsy vs. right temporal lobe epilepsy. In the right temporal lobe epilepsy group more contralateral connectivity can be seen compared to the left temporal lobe epilepsy group which corresponded with more contralateral neuropsychological deficits in this group. Figure adapted from Coito et al. (25). Permission for reuse granted by John Wiley and Sons (License Number 4518770594674). Spike-related network patterns in **(A)** LTLE and **(B)** RTLE.

Given the fact that M/ESI is increasingly validated to localize the irritative zone, using source connectivity will probably not add much information to localize the origin of the spike but could be used for mapping large-scale network aspects of interictal activity and their clinical diagnostic and prognostic correlates. Coito et al. studied the time-varying frequency specific directed connectivity between brain regions during spikes in temporal epilepsy using high density EEG (25). They found that the spike network in 16 temporal lobe epilepsy patients was more bilateral in right temporal epilepsy including some frontal connections as shown in Figure 3. Interestingly, this pattern was concordant with the neurocognitive profile of these patients showing more verbal and visuospatial alterations as well as impaired executive function (frontal) in right temporal lobe epilepsy at group level. These results are promising but limited to patients with a sufficient number of recorded spikes. So far, the clinical relevance of spike-related connectivity analysis regarding the risk of recurrence, drug treatment response, and prediction of post-operative outcome has not been assessed.

A DCM study on children with centrotemporal spikes found the strongest outflow in the central cortex, temporo-parietal junction and temporal pole with projections toward frontal regions and the contralateral hemisphere (69). The population chosen in this study unfortunately prevents invasive validation of this interesting approach based on a neural mass model.

### Non Spiking “Resting State” and Cognition

Apart from studying the brain during seizures or interictal “spiking” periods, there is an increasing interest to examine the “resting state” of the brain. Studies of resting-state connectivity could be interesting to understand more about the default state of the brain that might influence spike and seizure generation. In fact, there are several studies showing that network connectivity is altered in resting epileptic brains.

**Figure 4:**
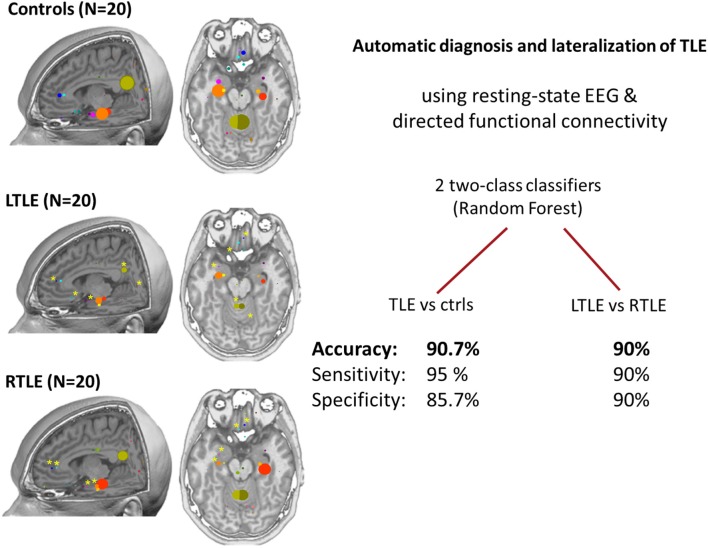
Patterns of connectivity in non-spiking high density EEG (24). **Left**: comparison between healthy controls, left temporal lobe epilepsy and right temporal lobe epilepsy (20 subjects in each group). In controls, posterior cingulate and medial temporal structures are strong drivers of the network, the maximum being in the posterior cingulate cortex. In patients, there is a global reduction of the drivers with the maximum located in the ipsilateral medial temporal structures at group level. **Right**: the use of machine learning (two random forest classifiers) allowed achieving very high accuracy for the prediction of individual subjects suggesting a role of this analysis as a biomarker (71). Permission for reuse granted by John Wiley and Sons (License Number 4518770257321).

High density EEG source connectivity identified the posterior cingulate cortex as the strongest driver in healthy subjects (70), while in 20 patients with temporal lobe epilepsy, the maximum outflow was in the ipsilateral medial temporal lobe (24). It needs to be considered that measures for connectivity that are frequency specific such as the partial directed coherence in Coito et al. (24) require selection of a frequency range of interest. In their study, Coito et al. (24) selected the alpha range, because the main drivers were found in this frequency band. The choice of the frequency band may contribute to variation of results across studies. However, a follow-up study used a classifier to distinguish between patients and controls with a high accuracy of 91% and to lateralize the focus in 90% of patients in the absence of visible EEG abnormality. This could offer a potential powerful diagnostic biomarker (71) (Figure 4). It needs to be mentioned, however, that classification analysis by use of machine learning has undergone significant change over the last years. Initial enthusiasm was often based on biased selection of characteristics (features) by which the groups of interest would be separated. The main problem consisted in inappropriate subset selection approaches, leading to overfitting the results to the analyzed sample, thus, great results of sensitivity and specificity, but poor generalizability. Verhoeven et al. (71) illustrate that iterative algorithms with a random selection procedure reveal instability in the selected features—illustrating the effect of fitting the model to the sample. To conclude, studies using machine learning techniques should always be interpreted by taking into account the feature subset selection algorithm and how cross-validation is done.

In generalized epilepsy (19 patients with drug naive juvenile myoclonic epilepsy), Clemens et al. performed source-based connectivity analysis from EEG recordings (19 electrodes) using correlation measures. They found increased alpha band connectivity and decreased beta band connectivity as well as larger integration compared to controls (72). These alterations occurred mostly in the somatosensory integration areas. Although not obtained with the same approach as the directed connectivity study in temporal lobe epilepsy described above, this study points out to a very different pattern of connectivity alterations that strengthen the potential diagnostic role of M/EEG source connectivity analysis for epilepsy classification in the absence of visible interictal activity.

However, some studies report increased connectivity (5, 7), and other studies report decreased connectivity (6, 73) or complex patterns of increased and decreased connectivity (24). It is likely that these differences are influenced by the type of epilepsy studied (genetic/idiopathic vs. focal/lesional epilepsy), clinical differences (e.g., seizure rate/freedom), the methods such as the choice of the frequency band, processing variants used and, possibly, medication (74). All these factors will need to be better addressed in future studies to allow better comparability. Currently, there is no established standard for this type of “resting-state” network analysis and there is only a limited understanding of the confounding technical and biological factors. Nevertheless, at least in some syndromes like idiopathic generalized epilepsy there is a spatial overlap between regions that show network alterations (3, 7) and spike sources (9) suggesting a biological link between them.

Under physiological conditions, the brain is constantly active, as is evident from EEG/MEG discharges even in the deepest stages of sleep. Moreover, the “resting state,” i.e., the status of the brain without an active external task, is not a homogenous, single state but rather a combination/interplay of different states. Indeed scalp EEG data can be decomposed into alternating stable scalp voltage topographies called “microstates,” (75). Future connectivity studies of the resting-state might gain from considering the different microstates separately. In these future studies, oversimplification of microstates to a few basic configurations is not recommendable. Larger scale evaluation of generalizability of microstates is first needed before choosing a set of pre-defined resting-state patterns. Moreover, comparison of microstates to the time-restricted view of source space connectivity is of interest, as time scale plays an important role in determining directed networks.

## Discussion

### What Have We Learned (so Far) From the Connectomes

#### Localization

For interictal epileptic activity and non-spiking periods, existing studies have not focused at localizing pathological activity but rather at describing the large-scale brain networks and the patterns of connections across individual patients and groups of patients vs. healthy subjects. It remains to be determined if the analysis of interictal connectivity provides an added value over “plain” source localization for estimating the epileptogenic zone and post-operative outcome. For seizure analysis, very promising localizing results have been found for ictal recordings, using high density as well as low density recordings in patients with good post-operative outcome. So far only retrospective ictal connectivity studies were done in limited and homogeneous cohorts. Prospective studies need to confirm these findings in larger groups of patients with temporal and extra-temporal lobe epilepsy and a variety of post-operative outcome to bring these techniques closer to clinical utility.

#### Diagnosis

The source connectivity during interictal spikes was shown to be concordant with cognitive deficits at group level (25). More contralateral spreading of the spikes was seen in right temporal lobe epilepsy compared to left temporal lobe epilepsy, which was in agreement with more contralateral neuropsychological deficits in right temporal lobe epilepsy. This is an indication that source connectivity has an additional potential diagnostic value. Nevertheless, the diagnostic added value should surpass the group level and be applicable to the individual patients before it can be used in patient treatment and follow-up.

There are promising results supporting connectivity analysis for improving the diagnosis of epilepsy and classification in the absence of visible EEG abnormalities (71). Further studies should include drug naive (first seizure) subjects, other types of epilepsies (generalized and focal) as well as patients with other neurological disorders (with and without structural abnormalities) including non-epileptic seizures. This would allow better estimating the sensitivity and specificity of such non-spiking EEG connectivity analysis that could assist the clinician in frequently difficult differential diagnoses. Indeed, abnormal connectivity patterns have been reported in 18 patients with non-epileptic seizures vs. controls (76). Abnormal findings based on high density EEG and phase-lag/synchronization measures affected mostly basal ganglia outflow although the ability of EEG connectivity to map connections from subcortical structures remains controversial. In patients with a first seizure, a connectivity study based on synchronization likelihood between scalp signals found that increased connectivity in the theta band was associated with an epilepsy diagnosis. In the absence of visible epileptic activity, connectivity could predict the diagnosis of epilepsy with sensitivity of 51% and specificity of 73% (77). In another study, increased theta band connectivity extracted from MEG in sensor space was shown to correlate with a higher number of epileptic seizures in brain tumor patients, indicating the potential to be used as biomarker for tumor-related epilepsy (78). Despite the methodological limitations related to scalp signal analysis, these studies pave the way for similar investigations on the diagnostic and prognostic added value of M/EEG connectivity analysis in source space. Also, connectivity results in source space might be more intuitive for the physician than in sensor space because connections between brain regions are easier to understand than connections between sensors that do not necessarily represent the connectivity of proximal brain regions.

#### Predicting Outcome

The benefits of interictal, ictal, or resting state connectivity as a predictor of disease evolution (recurrence after first seizure, response to drug treatment, or epilepsy surgery) have not been formally studied and the same needs for validation and comparison of methods discussed in the previous section also apply here.

Regarding vagal nerve stimulation, Wostyn et al. (79) studied source activity and connectivity of the P300 response with the vagal nerve stimulation system on/off and found that good response to therapy was correlated with specific patterns of source activity and connectivity, mostly involving the limbic system, insula, and the orbitofrontal region. However, the study only investigated EEG after implantation of the vagal nerve stimulator. It remains unclear if effective connectivity patterns can predict whether patients will respond or not to vagal nerve stimulation.

### Future Perspectives and Clinical Application

M/EEG source space connectivity techniques allow studying temporal patterns measured with M/EEG. For instance, it could be used to investigate network aspects of specific phenomena such as focal slowing [especially temporal, frontal, or occipital intermittent rhythmic delta activity (80)] and link specific patterns to specific forms of epilepsy (81). Furthermore, source space connectivity patterns could shed more light on how these M/EEG patterns are generated in the brain, and distinguish which patterns of focal slowing are potential surface correlates of epileptogenic activity from deeper regions.

#### Methodological Considerations

There exist a multitude of source localization methods and connectivity methods. It remains unclear which techniques should be used. Additional validation and comparison between source space connectivity methods is crucially needed in large patient cohorts with invasive validation. A pilot study showed that weighted minimum norm approach and phase-lag value were the best combination of inverse model and connectivity analysis on simulated data with application in only one patient (82). Such rigorous comparative approaches must be encouraged in broader clinical populations.

Several studies used informed selection of regions of interest in order to perform connectivity analysis, while others followed a rather data-driven approach. The drawback of the data-driven approach is that the selection of the interesting edges of the network is typically based on the magnitude of activity and/or interaction. However, the most meaningful regions do not necessarily exhibit the largest activity/connectivity with respect to the clinical question. For example, physiological process may exhibit a more prominent network than the pathological epileptic connection patterns. On this background, it is worth considering hypothesis driven analysis of source connectivity. However, in order to maintain objectivity, the grounds for selecting the regions of interest must be well motivated. One approach is to base the selection process on additional data. For example, resting-state fMRI or EEG-fMRI can be used to identify regions of interests that will be further investigated by MEG/EEG source connectivity (30). DCM, originally developed for fMRI, can also be understood as an informed approach and was earlier proposed to be used for estimating source space connectivity (83) and was used to determine the role of a-priori selected brain regions for seizure generation (84).

Currently, there is a lack of validation studies to bring source space connectivity into clinical practice. Even M/EEG source localization is still only performed in a modest number of centers worldwide, e.g., in the E-PILEPSY consortium electromagnetic source localization is only used at 12 out of 25 centers in the presurgical evaluation (85). Calculating source space connectivity is more complex than source localization alone. Given that the integration of source localization has already proven to be difficult in clinical practice, it can be anticipated that this will be an even larger challenge for source space connectivity. First, well designed prospective studies should show that source space connectivity has clear added value compared to the visual analysis of the electrophysiologist today on a patient specific level. Later, the methods should be available in standardized software with appropriate clearance for clinical use. Software for clinical use must come along with high usability, so that clinical-technical staff can do the necessary steps of processing (a) without risk of running into pitfalls that are common in source imaging and (b) within a workable time range, which is notoriously limited in clinical contexts. Next to this, standardized paradigms must be defined and tested, similarly to neuropsychological tests, in order to determine whether eloquent areas can be mapped by source connectivity analysis in pre-surgical evaluation. These paradigms must take into account the fact that network analyses require temporal information. Indeed, source-level connectivity needs different data properties than simple source-transformation of single events such as peaks of event related components. Only when all these limitations are overcome, can source connectivity be considered in clinical practice to assist in patient treatment and follow-up.

On the upper end of the EEG frequency range there are also promising fields of action for source connectivity: high frequency oscillations (HFOs), occurring above 80 Hz, have gathered great interest over the last two decades (86–88). However, evidence that HFOs might serve as an indicator for the region that needs to be resected in order to achieve seizure freedom is limited to invasive EEG recordings (89). MEG beamformer-based virtual sensors allowed to distinguish infrequent HFOs in MEG, from noise (90). It is of further interest whether measures of directed connectivity can inform us better about the very local propagation patterns of HFOs. Multiple potential clinical as well as basic science implications warrant the technical effort to approach this new perspective. For instance, the differential propagation patterns of HFOs might distinguish pathological from physiological HFOs on surface recordings—a problem that is hard to tackle so far (91).

A further crucial approach is the validation of the methods applied to EEG and MEG by concurrently recorded intracranial signals from regions deep within the brain. These studies offer unique validation opportunities, by providing direct evidence that the sources derived from surface recorded signals indeed correlate with the neuronal activities linked to specific cognitive functions recorded directly from the responsible brain areas. For instance, Crespo-García et al. (92) used simultaneous intracranial EEG and MEG and showed that hippocampal slow-theta activity was negatively correlated with spatial accuracy for memorized locations. Other studies mostly investigated the circumstances in which intracranial epileptic discharges can be detected with non-invasive recordings (93).Time-derived connectivity analysis and single-event analysis would critically depend on such simultaneous recordings for validation.

#### Brain Networks in Cognition

Epilepsy offers unique opportunities to examine the physiological foundations of human cognition when basic science is conducted in patients undergoing invasive recordings. Results obtained from surface EEG in healthy participants can be source-transformed, and the localization can serve to select patients undergoing epilepsy surgery according to their medically indicated placement of invasive electrodes. For example, Ponz et al. (94) identified disgust-effects in event-related potentials with generators in the left anterior insula, as localized by source transformation of 64-channel surface EEG in 21 healthy participants. Two patients suffering from left frontotemporal epilepsy underwent presurgical investigation with depth cortical electrodes. These case studies confirmed early emotion effects in insular and orbitofrontal electrodes when undergoing the same cognitive testing procedure. In another study, Dalal et al. (95) demonstrated the localizability of sources derived from both, MEG and EEG, obtained during self-paced finger movements to the sensorimotor cortex, where the localization was confirmed again by electrocorticography from two epilepsy patients. Selecting patients with specific combination of intracranial electrodes could also be used for investigating network characteristics and validating connectivity analysis in these cognitive processes.

Taking this approach into the other direction, knowledge obtained in basic cognitive neurosciences can inform the localization of eloquent areas for the surgical management of patients who are candidates for epilepsy surgery (96). Adequate design of cognitive paradigms to be used in order to activate the eloquent area is often based on prior research in healthy samples, as well as the validation of the respective analysis pipeline. For example, MEG sources for language localization and lateralization can guide preoperative decision making (97, 98). This technique, be it based on EEG or MEG, can easily be extended to other questions regarding the boundaries of eloquent and to-be resected tissue (99–101). However, there is no report regarding the additional information that could be gained by source-level connectivity. Coito et al. (25) reported that the difference in source-level network patterns during interictal spikes in right and left temporal lobe epilepsy overlapped with neuropsychological deficits. However, whether such cognitive correlates may be detectable also in the absence of spikes by EEG/MEG source-level connectivity needs to be determined in future studies. Research projects addressing source-level connectivity and cognition in epilepsy are highly warranted, as connections between eloquent areas and pathological regions may significantly contribute to the outcome if the pathological region or a crucial connection is targeted surgically.

## Conclusion

Source connectivity derived from EEG or MEG opens up new perspectives on the network disease epilepsy (see Box 1). We can more intuitively “see” the origin and spread of pathological or physiological activity and this information can be integrated into clinical decision making. Studies in limited cohorts have shown that source connectivity can be used to localize the epileptogenic zone from ictal epochs and interictal spikes, and that diagnosis of epilepsy from resting state M/EEG is feasible. Nevertheless, several obstacles need to be overcome to bring these techniques into clinical use: (i) source connectivity methods should be standardized and validated with respect to invasive recordings in large patient cohorts, (ii) software with appropriate clearance for clinical use that has high usability and requires limited time of the staff should be developed, (iii) prospective validation studies that show the added value of source connectivity over visual analysis need to be conducted in large heterogeneous patient cohorts, and (iv) standard paradigms and the respective analysis pipelines that allow to test functioning of eloquent areas need to be designed.

Box 1 **Summary Box**

EEG/MEG source imaging is performed on signals to project them into brain/source space.

Connectivity analysis can reveal epileptic networks, which show specific patterns:

- **ictal hyperconnectivity**: higher connectivity and, specifically, higher outgoing information flow from the epileptogenic zone (53, 54, 58) reflects (initial) spreading activation patterns of seizures.
- **interictal epileptic discharge propagation**: directed connectivity reveals propagation patterns of spikes and, thus, their origin (25, 68).
- **diagnosis in the absence of epileptiform activity**: source connectivity patterns distinguish patients from healthy controls (3, 24, 70–72, 76–78).

**Future directions** should address outcome prediction (79), model validation (82), concurrent recording with intracranial signals (92), standardized cognitive stimulation protocols for assessing the eloquent area, for bringing source connectivity analysis into clinical practice.

## Author Contributions

PvM provided the technical description of MEG/EEG source connectivity, ictal connectomes in epilepsy, combined contributions, and feedback by all authors and helped with the revision. YH described the background on connectivity, cognitive applications, future applications, and took care of the revision. NF provided insights into research and applications of MEG source connectivity, and clinical applications. SV summarized studies about interictal epileptiform discharges, detailed future perspectives, and clinical applications. All authors have edited, commented, and revised all sections of the manuscript and confirmed its final version.

### Conflict of Interest Statement

PvM is a co-founder and shareholder of Epilog NV (Ghent, Belgium). SV is an advisor and shareholder of Epilog NV (Ghent, Belgium). The remaining authors declare that the research was conducted in the absence of any commercial or financial relationships that could be construed as a potential conflict of interest.
